# External Validation of COOL-AF Scores in the Asian Pacific Heart Rhythm Society Atrial Fibrillation Registry

**DOI:** 10.1016/j.jacasi.2023.09.011

**Published:** 2023-11-14

**Authors:** Tommaso Bucci, Alena Shantsila, Giulio Francesco Romiti, Wee-Siong Teo, Tze-Fan Chao, Wataru Shimizu, Giuseppe Boriani, Hung-Fat Tse, Rungroj Krittayaphong, Gregory Y.H. Lip

**Affiliations:** a Liverpool Centre of Cardiovascular Science at University of Liverpool, Liverpool John Moores University and Liverpool Heart & Chest Hospital, Liverpool, UK; b Department of General and Specialized Surgery, Sapienza University of Rome, Rome, Italy; c Department of Translational and Precision Medicine, Sapienza University of Rome, Rome, Italy; d Department of Cardiology, National Heart Centre, Singapore; e Division of Cardiology, Department of Medicine, Taipei Veterans General Hospital, Taipei, Taiwan; f Institute of Clinical Medicine and Cardiovascular Research Center, National Yang Ming Chiao Tung University, Taipei, Taiwan; g Department of Cardiovascular Medicine, Nippon Medical School, Tokyo, Japan; h Department of Biomedical, Metabolic and Neural Sciences, Cardiology Division, University of Modena and Reggio Emilia, Policlinico di Modena, Modena, Italy; i Department of Medicine, Queen Mary Hospital, the University of Hong Kong, Hong Kong SAR, China; j Division of Cardiology, Department of Medicine, Faculty of Medicine Siriraj Hospital, Mahidol University, Bangkok, Thailand; k Danish Center for Health Services Research, Department of Clinical Medicine, Aalborg University, Aalborg, Denmark

**Keywords:** Asians, atrial fibrillation, bleeding, mortality, thromboembolism

## Abstract

**Background:**

The COOL-AF (Cohort of Antithrombotic Use and Optimal International Normalized Ratio Levels in Patients with Atrial Fibrillation) risk scores for death, bleeding, and thromboembolic events (TEs) were derived from the COOL-AF cohort from Thailand and require external validation.

**Objectives:**

The authors sought to externally validate the COOL-AF scores in the APHRS (Asia-Pacific Heart Rhythm Society) registry and to compare their performance in the ESC-EHRA (European Society of Cardiology-European Heart Rhythm Association) EORP-AF (EURObservational Research Programme in Atrial Fibrillation) General Long-Term Registry.

**Methods:**

We studied 3,628 APHRS and 8,825 EORP-AF patients. Receiver operating characteristic (ROC) curves and Cox regression analyses were used to test the predictive value of COOL-AF scores and to compared them with the CHA_2_DS_2_-VASc and HAS-BLED scores.

**Results:**

Patients in the EORP-AF were older, had a higher prevalence of male sex, and were at higher thromboembolic and hemorrhagic risk than APHRS patients. After 1 year of follow-up in APHRS and EORP-AF, the following events were recorded: 87 (2.4%) and 435 (4.9%) death for any causes, 37 (1.0%) and 111 (1.3%) major bleeding, and 25 (0.7%) and 109 (1.2%) TEs, respectively. In APHRS, the COOL-AF scores showed moderate-to-good predictive value for all-cause mortality (area under the curve [AUC]: 0.77; 95% CI: 0.71-0.83), major bleeding (AUC: 0.68; 95% CI: 0.60-0.76), and TEs (AUC: 0.61; 95% CI: 0.51-0.71), and were similar to the CHA_2_DS_2_-VASc and HAS-BLED scores. In EORP-AF, the predictive value of COOL-AF for all-cause mortality (AUC: 0.68; 95% CI: 0.65-0.70) and major bleeding (AUC: 0.61; 95% CI: 0.60-0.62) was modest and lower than in APHRS. In EORP-AF, the COOL-AF score for TE was inferior to the CHA_2_DS_2_-VASc score.

**Conclusions:**

The COOL-AF risk scores may be an easy tool to identify Asian patients with AF at risk for death and major bleeding and performs better in Asian than in European patients with AF. (Clinical Survey on the Stroke Prevention in Atrial Fibrillation in Asia [AF-Registry]; NCT04807049)

Atrial fibrillation (AF) is the most common arrhythmia worldwide and is associated with an increased risk of systemic embolism and death.^1^ Oral anticoagulation (OAC) with vitamin K antagonists (VKAs) or non-vitamin K anticoagulants (NOACs), has been shown to reduce thromboembolic events (TEs) and to improve survival in patients with AF; however, the use of OAC has to balance reduction of stroke against the potential risk of bleeding events.^2^

Several risk-stratification scores have been proposed to stratify the thrombotic and hemorrhagic risk in patients with AF; the most commonly used clinical practices are the CHA_2_DS_2_-VASC and HAS-BLED scores.^3^^,^^4^ Despite these scores having good predictive value and their recommendation by international guidelines,^5^, ^6^, ^7^ they have been derived from studies mainly performed in Western countries, and their application in Asian populations has been debated. Indeed, compared with Western patients, Asian patients with AF have a higher predisposition to bleeding and a different TE risk profile, characterized by a higher prevalence of heart failure (HF) and diabetes and a lower prevalence of vascular disease.^8^, ^9^, ^10^

Recently, 3 new predictive risk models to identify patients with AF at high risk of death for any causes, major bleeding, and TEs have been proposed from the COOL-AF (Cohort of Antithrombotic Use and Optimal International Normalized Ratio [INR] Level in Patients With Nonvalvular Atrial Fibrillation in Thailand) study.^11^ In these patients, the COOL-AF scores showed more improved performances than the CHA_2_DS_2_-VASc and HAS-BLED scores and have been proposed as possible Asian-specific risk scores for all-cause mortality, major bleeding, and TEs.^11^ These risk scores require external validation in other Asian and non-Asian cohorts.

The aim of this study was to validate the COOL-AF score externally in the APHRS (Asia-Pacific Heart Rhythm Society) registry and to compare its performance in the ESC-EHRA (European Society of Cardiology-European Heart Rhythm Association) EORP-AF (EURObservational Research Programme in Atrial Fibrillation) general long-term registry.

## Methods

The study protocol for patient selection and data collection for the APHRS and EORP-AF were similar, as reported previously.^12^^,^^13^ In brief, the APHRS registry was started in 2015, and the enrollment finished in 2017. The population was composed of consecutive inpatients and outpatients with AF who had undergone cardiology examinations in tertiary and general hospitals in 5 Asian countries (Hong Kong, South Korea, Japan, Singapore, and Taiwan). All eligible patients had electrocardiogram (ECG)-documented AF within 12 months before their enrollment visits and had signed written informed consent forms according to the local regulations. After the baseline clinical assessment, the 1-year follow-up was performed by the local investigators. The study protocol was approved by local ethics committees, and the trial was registered on ClinicalTrials.gov (NCT04807049).

### Study outcomes

Adverse outcomes were registered after 1 year of follow-up observation. The primary endpoints of the study were all-cause mortality, major bleedings (including intracranial hemorrhage and extracranial major bleedings) and TEs (including stroke, transient ischemic attack [TIA], and any peripheral embolism).

### Risk scores

The CHA_2_DS_2_-VASc score was calculated as follows: congestive HF (1 point); hypertension (1 point); age 65 to 74 (1 point) and >75 years (2 points); diabetes (1 point); stroke (2 points); vascular disease (1 point); and female sex category (1 point). Patients with CHA_2_DS_2_-VASc ≥2 were considered at high-risk for TEs.^5^ The HAS-BLED score was calculated as follows: uncontrolled hypertension (1 point), abnormal renal, or liver function (1 point); history of stroke (1 point); history of bleeding (1 point); labile INR (1 point); age >65 years (1 point); and drugs (eg, aspirin or nonsteroidal anti-inflammatory drugs or alcohol) (1 point). Patients with HAS-BLED ≥3 were considered at high risk for bleeding.^5^ In APHRS, the HAS-BLED score was calculated by the investigators (including the labile INR criterion, when applicable) and reported in the case records.

The COOL-AF scores were calculated as was previously reported^14^:

• COOL-AF score for all-cause mortality at 1-year was calculated as follows: 1 − 0.94712516^exp (Prognostic Index)^ when prognostic index = 0.020319 · age − 0.087589 · body mass index (BMI) − 0.456114 · paroxysmal AF + 0.518448 · bleeding + 0.805105 · anemia.
• COOL-AF score for major bleeding at 1 year was calculated as follows: 1 − 0.99950939 ^exp (Prognostic Index)^ when prognostic index = 0.042377 · age − 0.512419 · female + 1.679421 · renal replacement therapy + 0.601297 · anemia + 0.849478 · OAC.
• COOL-AF score for TE 1 year was calculated as follows: 1 − 0.99501052 ^exp (Prognostic Index)^ when prognostic index = 0.841293 · hypertension + 1.326225 · chronic kidney disease (CKD) − 0.716626 · OAC.

The COOL-AF score components were defined according to the COOL-AF original study as follows: Anemia was defined according to the World Heart Organization criteria as a hemoglobin level <13 g/dL for male subjects and <12 g/dL for female subjects^15^; CKD was defined as an estimated glomerular function <60 mL/min/1.73 m^2^, according to the Kidney Disease Improving Global Outcomes (KIDGO) guidelines.^16^ Previous bleeding was defined as the occurrence of major bleeding or clinically relevant non-major bleeding according to the International Society on Thrombosis and Haemostasis (ISTH) guidelines.^17^^,^^18^ Paroxysmal AF and hypertension diagnosis were determined according to the ESC recommendations.^5^^,^^19^

### Statistical analyses

Continuous variables are reported as median(IQR), whereas categorial variables are reported as percentages. Comparison among groups has been done with a nonparametric test (Mann-Whitney test) and chi-square test. Receiver operating characteristic (ROC) curves were used to assess the ability of the COOL-AF scores to predict the primary endpoints. Comparisons of the predictive ability of COOL-AF scores with CHA_2_DS_2_-VASc for all-cause of mortality and TEs and HAS-BLED for major bleeding were performed in each population by ROC pairwise comparison. Area under the curve (AUC) values were calculated using the method described by Delong et al.^14^ In addition, we estimated the clinical usefulness and net benefit of COOL-AF scores and CHA_2_DS_2_-VASc or HAS-BLED scores using the decision curve analysis (DCA) with the method proposed by Vickers et al.^20^

In each population, we used the ROC curve with Youden’s J statistic (J index) to find the specific optimal cutoff to dichotomize the COOL-AF scores.

J Index was calculated as follows: sensitivity/specificity-1 = (true positives/true positives + false negatives) + (true negatives/true negatives + false positives)-1

Plots of Kaplan-Meier curves for time to all-cause mortality, major bleeding, and any TE according to the dichotomized COOL-AF scores were performed. Survival distributions were compared using the log-rank test. Cox proportional hazards regression time to the first event analysis was used to calculate adjusted relative HRs and 95% CI of outcomes. All the multivariable Cox regression analyses, performed to investigate the association between COOL-AF scores and the primary outcomes, were adjusted for the following covariates: age, sex, and CHA_2_DS_2_-VASc ≥2 or HAS-BLED ≥3. Only a 2-sided *P* value <0.05 was considered statistically significant. Patients without available data to calculate the COOL-AF scores or follow-up were excluded from the analysis. All statistics were performed by SPSS statistical software, version 25.0 (IBM SPSS Statistics), and MedCalc (MedCalc Software Ltd).

## Results

Of the 4,666 and 11,096 patients enrolled in the APHRS and EORP-AF, the total number of patients with all the data needed to calculate COOL-AF scores and available 1-year follow-up was 3,628 (77.7%) and 8,825 (79.5%), respectively. APHRS patients not included in this analysis were younger, with a higher prevalence of male sex, and a lower risk for TEs compared with included patients. EORP-AF patients not included were older, but no significative other differences were found for thrombotic and hemorrhagic baseline risks.

As reported in Table 1, EORP-AF patients were older; had a higher prevalence of female patients; and a higher median value of systolic and diastolic blood pressure, heart rate, and BMI. Regarding the clinical history, EORP-AF patients showed a higher prevalence of HF, coronary artery disease (CAD), stroke and TIA, dyslipidemia, chronic obstructive pulmonary disease, and CKD, whereas APHRS patients had a higher prevalence of paroxysmal AF, previous bleeding, diabetes, and anemia. EORP-AF patients were at higher risk of both thrombotic and hemorrhagic events, as showed by the higher prevalence of patients with CHA_2_DS_2_-VASc ≥2 and HAS-BLED ≥3. EORP-AF patients were more often prescribed with antithrombotic treatment and showed a higher use of both antiplatelet and OAC. The most used OACs were VKAs in EORP-AF and non-VKA oral anticoagulants (NOACs) in APHRS.

**Table 1:** Baseline Characteristics of Study Populations

	APHRS (n = 3,628)	EORP-AF (n = 8,825)	*P* Value
Age (y)	68.5 (61.0-76.0)	71.0 (63.0-77.0)	<0.001
Female	33.4 (1,210)	40.1 (8,358)	<0.001
BMI (kg/m^2^)	24.7 (22.3-27.3)	27.5 (24.8-31.1)	<0.001
SBP (mm Hg)	128 (117-140)	130 (120-142)	<0.001
DBP (mm Hg)	74 (66-82)	80 (70-87)	<0.001
HR (beats/min)	75 (66-85)	78 (66-92)	<0.001
Paroxysmal AF	42.0 (1,520)	26.2 (2,308)	<0.001
HF	20.8 (749)	38.3 (3,355)	<0.001
CAD	19.5 (695)	27.8 (2,339)	<0.001
Stroke/TIA	9.8 (355)	8.6 (754)	0.026
Bleeding	7.5 (271)	5.4 (476)	<0.001
Diabetes	24.8 (891)	22.8 (2,007)	0.021
Hypertension	61.4 (2,218)	61.8 (5,457)	0.668
Smoking	8.8 (320)	9.4 (778)	0.326
Dyslipidemia	39.1 (1405)	41.9 (3,563)	0.005
Dementia	1.5 (54)	1.1 (95)	0.055
COPD	2.6 (95)	8.7 (759)	<0.001
CKD	7.5 (271)	12.3 (1,084)	<0.001
Dialysis	1.2 (44)	0.4 (37)	<0.001
Anemia	6.6 (241)	5.0 (437)	<0.001
CHA_2_DS_2_-VASc ≥2	72.6 (2,634)	80.5 (7,102)	<0.001
HAS-BLED ≥3	14.2 (516)	17.4 (1,532)	<0.001
Antiplatelet	15.0 (543)	18.3 (1,617)	<0.001
OAC	3,016 (83.1)	86.7 (7,654)	<0.001
VKA	20.0 (725)	50.5 (4,455)	<0.001
NOAC	63.1 (2,291)	36.3 (3,205)	<0.001
Beta-blocker	51.6 (1,861)	68.8 (2,751)	<0.001
CCB	23.4 (848)	16.9 (1,490)	<0.001
Statin	37.8 (1,363)	42.3 (3,730)	<0.001
ACEI/ARB	39.5 (1,428)	60.5 (5,326)	<0.001

Values are median (IQR) or % (n).

ACEI = ACE inhibitor; AF = atrial fibrillation; APHRS = Asian Pacific Heart Rhythm Society; ARB = angiotensin receptor blocker; BMI = body mass index; bpm = beats per minute; CAD = coronary artery disease; CCB = calcium channel blocker; CKD = chronic kidney disease; COPD = chronic obstructive pulmonary disease; DBP = diastolic blood pressure; EORP-AF = EURObservational Research Programme in Atrial Fibrillation; HF = heart failure; HR = heart rate; NOAC = non-vitamin K anticoagulant; OAC = oral anticoagulation; SBP = systolic blood pressure; TIA = transient ischemic attack; VKA = vitamin K antagonist.

### Prediction of mortality, bleeding, and thromboembolism

**Figure 1:**
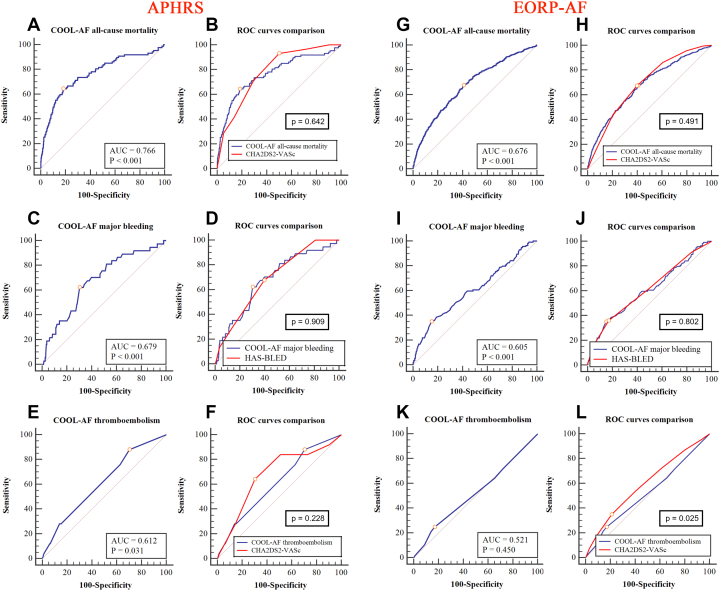
Receiver Operating Curves for COOL-AF Scores COOL-AF score for all-cause mortality (A) and its comparison with CHA_2_DS_2_-VASC (B); COOL-AF score for major bleeding (C) and its comparison with HAS-BLED (D); COOL-AF score for thromboembolism (E) and its comparison with CHA_2_DS_2_-VASc (F) in APHRS. COOL-AF score for all-cause mortality (G) and its comparison with CHA_2_DS_2_-VAS_C_ (H), COOL-AF score for major bleeding (I) and its comparison with HAS-BLED (J); COOL-AF score for thromboembolism (K) and its comparison with CHA_2_DS_2_-VASc (L) in EORP-AF. APHRS = Asian Pacific Heart Rhythm Society; COOL-AF = Cohort of Antithrombotic Use and Optimal International Normalized Ratio Levels in Patients With Atrial Fibrillation; EORP-AF = EURObservational Research Programme in Atrial Fibrillation.

In APHRS, the COOL-AF scores for all-cause mortality and major bleeding showed moderate-to-good predictive value (AUC: 0.77; 95% CI: 0.71-0.83; *P* < 0.001 and AUC: 0.68; 95% CI: 0.60-0.76; *P* < 0.001, respectively) (Figures 1A and 1C), and AUCs were nonstatistically significant different from those of CHA_2_DS_2_-VASc (AUC: 0.78; 95% CI: 0.74-0.82; *P* = 0.642) (Figure 1B) and HAS-BLED (AUC: 0.69; 95% CI: 0.61-0.76; *P* = 0.909) (Figure 1D) scores. The COOL-AF score for TEs showed a low predictive value (AUC: 0.61; 95% CI: 0.51-0.71; *P* = 0.031) (Figure 1E), with no statistically significant differences with CHA_2_DS_2_-VASc score (AUC: 0.68; 95% CI: 0.57-0.79; *P* = 0.228) (Figure 1F).

In EORP-AF, the COOL-AF scores for all-cause mortality and major bleeding showed a moderate predictive value (AUC: 0.68; 95 % CI: 0.65-0.70; *P* < 0.001 and AUC: 0.61, 95% CI: 0.60-0.62; *P* = 0.001, respectively) (Figures 1G and 1I) with no statistically significant differences compared with CHA_2_DS_2_-VASc (AUC: 0.69; 95% CI: 0.66-0.71, 0.491; *P* = 491) (Figure 1H) and HAS-BLED scores (AUC: 0.61; 95% CI: 0.56-0.67; *P* = 0.802) (Figure 1J). COOL-AF for TEs was nonsignificant (AUC: 0.52; 95% CI: 0.47-0.58; *P* = 0.450) (Figure 1K) and inferior to CHA_2_DS_2_-VASc (AUC: 0.59; 95% CI: 0.54-0.65; *P* = 0.025) (Figure 1L).

For the specific optimal cutoff to dichotomize the COOL-AF scores, the J indexes identified in APHRS and EORP-AF for each COOL-AF score were, respectively, –0.024122609 and –0.0553404034 for COOL-AF all-cause mortality; 0.001869861 and 0.001995343 for COOL-AF major bleeding; and –0.003590973 and 0.000623386 for COOL-AF TEs. The prevalence of patients with COOL-AF score > J index in APHRS and EORP-AF was 25.4% (n = 922) and 45.9% (n = 4,049) for COOL-AF all-cause mortality; 30.7% (n = 1,112) and 17.2% (n = 1,514) for COOL-AF major bleeding; and 70.5% (n = 2,558) and 65.3% (n = 5,762) for COOL-AF TEs.

### Survival analysis

The median follow up was 365 (IQR: 343-373) days in APHRS and 367 (IQR: 357-383) days in EORP-AF. During the 1-year follow-up, in APHRS and EORP-AF the following events were recorded: 87 (2.4%) and 435 (4.9%) death for any causes, 37 (1.0%) and 111 (1.3%) major bleeding, and 25 (0.7%) and 109 (1.2%) TEs, respectively.

**Figure 2:**
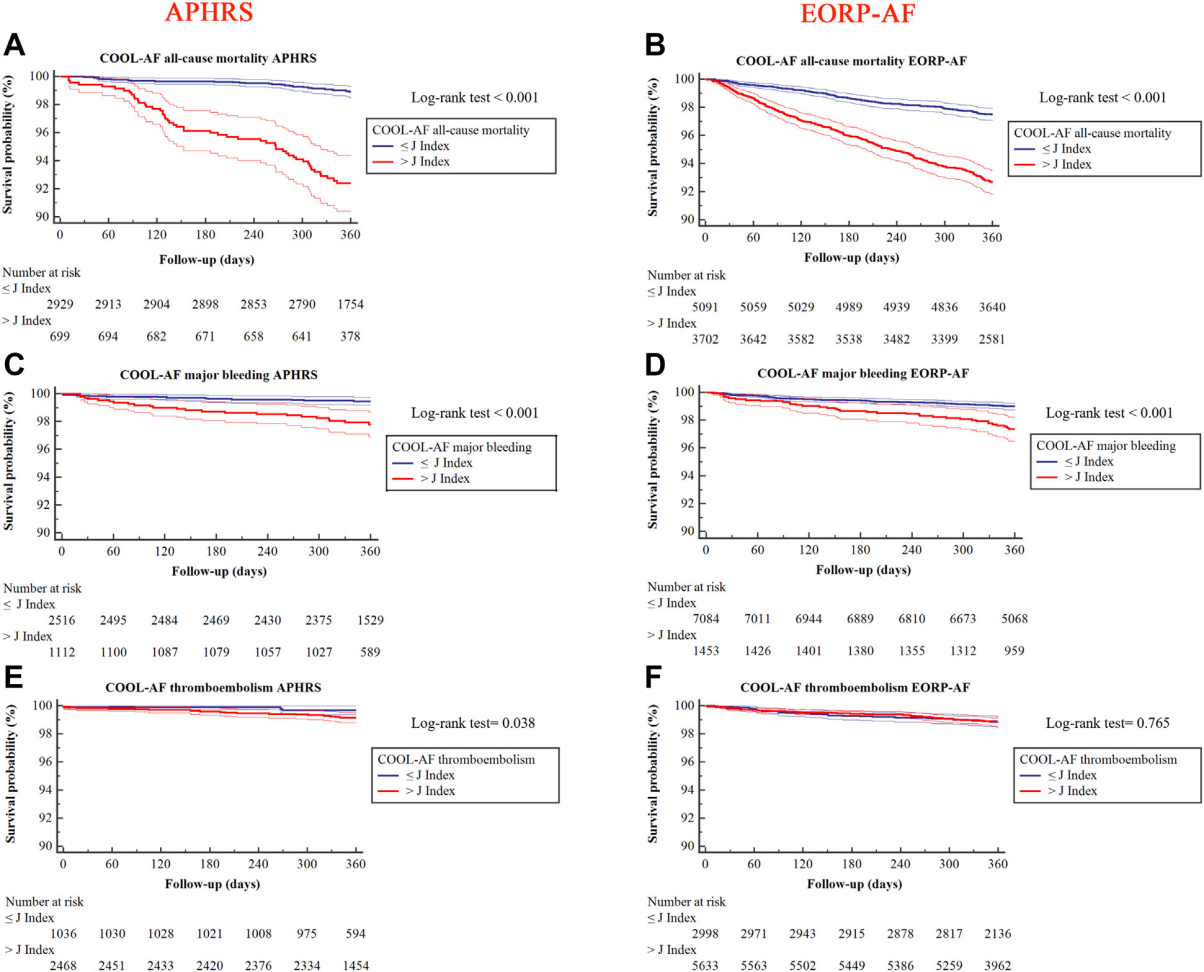
Multivariate Cox-Regression Analysis for COOL-AF Scores COOL-AF all-cause mortality in APHRS (A) and EORP-AF (B), COOL-AF major bleeding in APHRS (C) and EORP-AF (D), COOL-AF thromboembolism in APHRS (E) and EORP-AF (F). Abbreviations as in Figure 1.

In APHRS patients with COOL-AF scores > J index, the 1-year incidence of all-cause mortality, major bleeding, and TEs was higher than patients ≤ J index (6.4% [n =59] vs 1.0% (n = 28), log-rank test <0.001; 2.1% (n = 23) vs 0.6% (n = 14), log-rank test <0.001; and 0.9% (n = 22) vs 0.6% (n = 3), log-rank test = 0.038, respectively) (Figures 2A, 2C, and 2E). In EORP-AF, in patients with COOL-AF all-cause mortality and major bleeding scores > J index, the 1-year incidence of all-cause mortality and major bleeding was higher than patients ≤ J index (7.3% [n = 297] vs 2.9% [n = 138], log-rank test <0.001; and 2.7% [n = 41] vs 1.0% [n = 70], log-rank test <0.001; respectively) (Figures 2B and 2D), whereas no significative difference was found for COOL-AF TEs (1.2% [n = 70] vs 1.3% [n = 39], log-rank test = 0.765) (Figure 2F).

### Cox regression models

The dichotomized COOL-AF scores were tested in different Cox regression models adjusted for age, sex, and CHA_2_DS_2_-VASc ≥2 or HAS-BLED≥3 (Table 2). COOL-AF score for all-cause mortality was significantly associated with all-cause mortality after adjustment for age, sex, and CHA_2_DS_2_-VASc ≥2 in both APHRS (HR: 4.35; 95% CI: 2.65-7.14) and EORP-AF (HR: 1.70; 95% CI: 1.36-2.14) (Table 2, Model A). COOL-AF major bleeding was significantly associated with major bleeding after adjustment for age, sex and HAS-BLED≥3 in both APHRS (HR: 3.41; 95% CI: 1.32-8.80) and EORP-AF (HR: 2.52; 95% CI: 1.56-3.46) (Table 2, Model B). COOL-AF TE was not associated with TEs after adjustment for age, sex, and CHA_2_DS_2_-VASc ≥2 in both populations (Table 2, Model C).

**Table 2:** Predictive Models for COOL-AF Scores in APHRS and EORP-AF

	APHRS			EORP-AF		
	HR	96% CI	*P* Value	HR	96% CI	*P* Value
Model A						
Age (continuous)	1.04	1.01-1.07	0.004	1.06	1.04-10.7	<0.001
Female	0.93	0.61-1.44	0.758	0.88	0.72-1.08	0.219
COOL-AF score all-cause mortality > J index	4.35	2.65-7.14	<0.001	1.70	1.36-2.14	<0.001
CHA_2_DS_2_-VASc ≥2	3.50	1.18-12.06	0.047	1.95	1.16-3.64	0.011
Model B						
Age (continuous)	1.02	0.97-1.06	0.461	1.00	0.98-1.03	0.704
Female	1.49	0.69-3.20	0.310	1.23	0.80-1.90	0.350
COOL-AF score major bleeding > J index	3.41	1.32-8.80	0.011	2.52	1.49-4.25	0.001
HAS-BLED ≥3	2.56	1.26-5.18	0.009	2.32	1.56-3.46	<0.001
Model C						
Age (continuous)	1.05	1.01-1.10	0.022	1.02	0.99-1.04	0.151
Female	1.42	0.63-3.22	0.404	1.22	0.83-1.80	0.309
COOL-AF score thromboembolism > J index	3.14	0.90-10.93	0.073	1.50	0.97-2.34	0.071
CHA_2_DS_2_-VASc ≥2	0.62	0.16-2.43	0.493	1.46	0.71-2.99	0.301

Abbreviations as in Table 1.

### Discrimination analyses

We tested the clinical usefulness and net clinical benefit of the 3 COOL-AF scores using DCA (Supplemental Figures 1 and 2). The comparison between COOL-AF all-cause mortality and CHA_2_DS_2_-VASc showed marginal improvement in net clinical benefit in APHRS (but not EORP-AF), but the COOL-AF major bleeding and HAS-BLED and COOL-AF TE and CHA_2_DS_2_-VASc showed overlapping curves in both populations.

## Discussion

In this study, we have performed the first external validation of the COOL-AF scores, testing their predictive value in a large prospective cohort of Asian patients with AF and comparing their performance with Europeans. We found that all the COOL-AF scores are significantly associated with the primary outcomes in Asian patients with AF at risk for death and major bleeding and seems to perform better in Asian patients with AF compared with Europeans (Central Illustration).

**Central Illustration:**
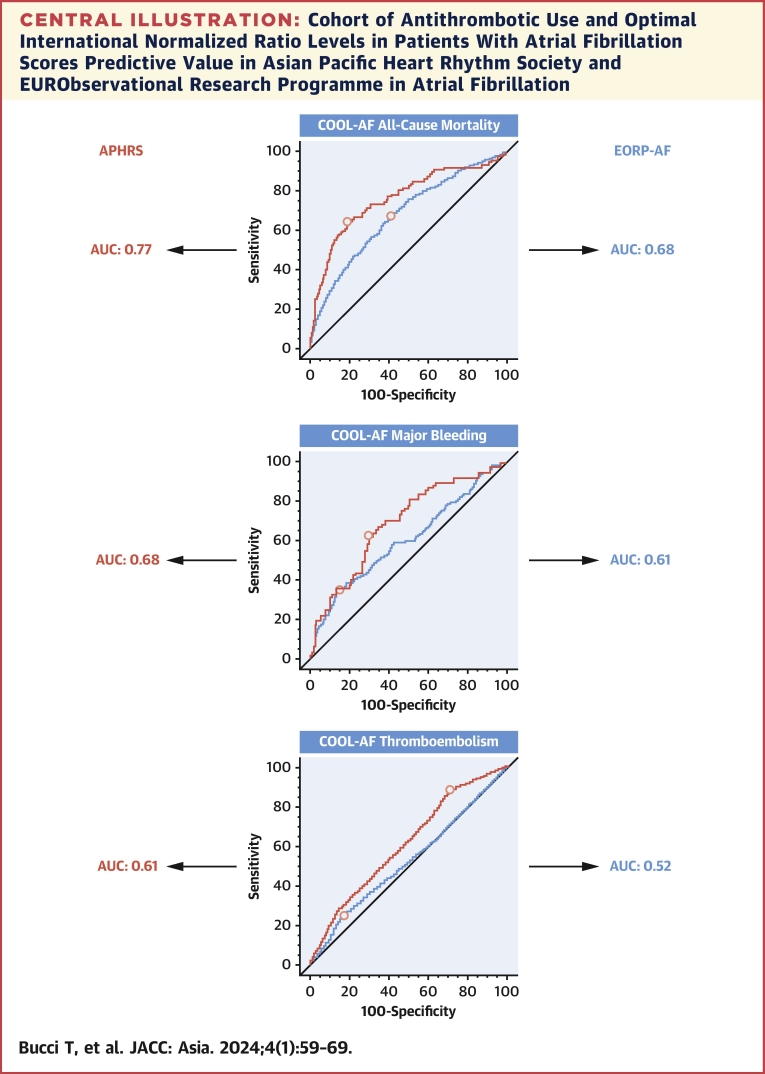
Cohort of Antithrombotic Use and Optimal International Normalized Ratio Levels in Patients With Atrial Fibrillation Scores Predictive Value in Asian Pacific Heart Rhythm Society and EURObservational Research Programme in Atrial Fibrillation The COOL-AF scores for all-cause mortality and major bleeding had good predictive value in both the registries, whereas the COOL-AF score for thromboembolism had a modest predictive value only in APHRS. Overall, the COOL-AF scores generally performed better in Asian than in European patients with AF. APHRS = Asian Pacific Heart Rhythm Society; AUC = area under the curve; AF = atrial fibrillation; COOL-AF = Cohort of Antithrombotic Use and Optimal International Normalized Ratio Levels in Patients With Atrial Fibrillation.

The predictive value of COOL-AF score for all-cause mortality in APHRS (AUC: 0.77) was similar to that reported in the COOL-AF study (AUC: 0.73),^11^ had a moderately good performance also in EORP-AF (AUC: 0.66), and was independent and similar to the CHA_2_DS_2_-VASc score in both populations. Of note, the COOL-AF score for all-cause mortality is calculated with several factors not included in the CHA_2_DS_2_-VASc score, and these results take into account additional variables such as BMI, history of bleeding, anemia, and the type of AF, in addition to the traditional risk factors, leading to better death risk stratification in Asian patients with AF. This may be of importance in Asian patients in whom the association among BMI, body mass composition, and health risk is different from Europeans.^21^ Indeed, a recent study in Korean patients with AF has shown that each BMI increase of 5 kg/m^2^ was associated with lower risks of ischemic stroke (HR: 0.89; 95% CI: 0.80-0.99), major bleeding (HR: 0.79; 95% CI: 0.69-0.92), and all-cause mortality (HR: 0.66, 95% CI: 0.60-0.72).^22^ Furthermore, a very low BMI may be associated with alterations in OAC metabolism that could be responsible for bleeding episodes and anemia.^23^^,^^24^ Of note, the COOL-AF score for all-cause mortality include the AF pattern and consider paroxysmal AF as a factor associated with lower TE and mortality risk when compared with persistent and permanent AF.^25^

In our study, the incidence of major bleeding was higher in EORP-AF patients than in the APHRS cohort. These results, besides the higher baseline HAS-BLED score in EORP-AF, should be also related to the different type of OAC used in these 2 populations. The most used OAC treatment was VKAs in EORP-AF and NOACs in APHRS patients. Several studies showed that NOAC treatment compared with VKA treatment is associated with an absolute lower risk of bleeding and that the magnitude of this reduction was more pronounced in Asian compared with non-Asian patients.^26^ Even in patients only treated with NOACs, the risk of intracranial bleeding is still numerically higher in Asians than Western patients,^27^, ^28^, ^29^, ^30^ showing that a specific Asian risk score for bleeding is still needed. The predictive value of COOL-AF score for major bleeding in APHRS (AUC: 0.68) was similar to that reported in the COOL-AF study (AUC: 0.71),^11^ but had a lower predictive role in EORP-AF (AUC: 0.61) and was independent and similar to the HAS-BLED risk score in both the populations.

These results suggest the importance of detecting anemia in patients with AF to stratify the risk of all-cause mortality and major bleeding. This point is in accordance with 2 previous studies in 4,824 Chinese and 1,562 Thai patients with AF in which anemia was an independent risk factor for major bleeding, HF, and death^31^^,^^32^ but is in contrast to a recent study on 15,606 Chinese patients in which anemia was independently associated with all-cause death, cardiovascular death, but not with major bleeding.^33^ The conflicting results from studies investigating the associations between anemia and major bleeding in AF are probably caused by several factors: In Asia, the proportion of patients on OAC differs considerately in the different areas influencing the hemorrhagic risk; no information about the type of anemia was reported making impossible to understand its clinical weight; and no study was designed to clarify if the lower hemoglobin is a result rather than a cause of bleeding.

The COOL-AF score for major bleeding considers female sex as a possible protective factor for hemorrhagic events and could represent another possible explanation to its independency from HAS-BLED. The protective value of female sex for major bleeding in the COOL-AF study is supported by growing evidence that female sex is a Janus-faced factor associated with a higher risk of systemic embolism and lower risk of bleeding than male patients.^3^^,^^34^^,^^35^

In our study, the predictive value of COOL-AF score for TEs was significantly lower (AUC: 0.61) than that reported in the COOL-AF study (AUC: 0.70). In APHRS, COOL-AF for TEs showed a low predictive value and was similar to CHA_2_DS_2_-VASc, whereas in EORP-AF, it showed no association with TE, and its performance was inferior to CHA_2_DS_2_-VASc. When interpretating this result, some possible confounding factors should be considered. In the COOL-AF score for TE, 1 of the strongest risk factors is represented by CKD. In the APHRS registry, we found a 7-fold lower prevalence of CKD (7.5% vs 51.6%) and a significantly lower 1-year incidence of TEs (1.4 of 100 patients per year vs 0.7 of 100 patients per year) than the COOL-AF study, which could be associated with a loss of statistical power in the current study. The lack of any predictive value of COOL-AF score for TE in EORP-AF seems to confirm its low performance, underlying that the determinants of the thrombotic risk in Europeans may be different from that seen in Asians.

To date, although growing evidence showed the presence of ethnic-specific factors in determining the risk of death, bleeding, and thromboembolic events in patients with AF, the international guidelines have not still adopted or proposed any approach that considers these aspects.^5^, ^6^, ^7^ The APHRS guidelines for management of AF in Asians underline the importance of preferring NOACs to VKAs for prevention of stroke because of the higher predisposition to bleeding in those patients, but do not provide any clear indication regarding the possibility to consider ethnic-specific factors to characterize the risk of adverse events.^6^ Although the CHA_2_DS_2_-VASc and HAS-BLED risk scores represent the best predictive tools to identify high-risk patients, the management of patients classified as low-risk is still debated.^36^ In these patients, a regular risk (re)assessment has been proposed to detect the onset of incident cardiovascular risk factors early that can drive therapeutic decisions.^37^ In this context, adding to the information given by the classical risk scores, such as those derived from the COOL-AF scores, could potentially help the clinician to better stratify the risk of adverse events in Asian patients with AF. Furthermore, in the era of a more holistic or integrated care approach to management of AF, the spread of this concept to the ethnic-associated factors could lead to a further improvement of the short- and long-term outcomes.

### Study limitations

The observational nature limits the strength of the evidence derived from this study. The presence of baseline differences between Asian and European patients could have influenced the predictive performance of the COOL-AF scores. Furthermore, given the high degree of heterogeneity within the different Asian (and European) geographic areas considered in this study, we cannot exclude the effect of ethnic and geographic factors in the performance of the COOL-AF scores. However, the main clinical and demographic characteristics of our APHRS population are similar to those reported in other Asian-based AF cohorts from China^38^ and Japan,^39^ allowing us to consider it as a representative Asian cohort of patients with AF.

Given the relatively low sample size of the study, when stratifying results by countries that participated in the APHRS registry, we were unable to analyze whether the performance of the COOL-AF scores differ among recruiting countries as well as to implement newer techniques (such as machine-learning algorithms), which may improve our ability to predict prognosis and major outcomes; further studies with larger sample size are therefore needed to answer these open questions. The low mortality rate observed in the APHRS registry is unlikely to affect the risk of competitive events, whereas the 1-year risk of outcomes was assessed by Cox proportional hazards regression time to the first event analysis, and no competing risk models for multiple events were used.

Finally, some of the variable needed for COOL-AF scores, such as anemia and CKD, are dynamic; as the data were collected at baseline, we cannot exclude that some patients with mild anemia or CKD ameliorate their status or that some patients with barely normal hemoglobin or creatinine values got worse during the observation period. Although we considered approximately the 80% of the initial cohort in both registries, the clinical differences between patients included and excluded from this analysis could have introduced some selection bias.

## Conclusions

The COOL-AF risk scores may be an easy tool to identify Asian patients with AF at risk for all-cause mortality and major bleeding and seems to perform better in Asian patients than in European patients with AF.

Perspectives **COMPETENCY IN PATIENTS CARE AND PROCEDURAL SKILLS:** The COOL-AF scores have been proposed as Asian predictive models for patients with AF. The different predictive value in Asians and Europeans suggest that the risk of death, major bleeding, and thromboembolic events is influenced by ethnic-specific risk factors.

**TRANSLATIONAL OUTLOOK:** Further studies are needed to better characterize the specific weight of each cardiovascular risk factor in determining the death, bleeding, and thrombotic-risk profile in different ethnic groups.

## Funding Support and Author Disclosures

Dr Romiti has done consultancy for Boehringer Ingelheim and has received an educational grant from Anthos, outside the submitted work; no fees are directly received personally. Dr Shimizu has received grants from Daiichi Sankyo Co, Ltd and Nippon Boehringer Ingelheim Co, Ltd; and remuneration for lectures, presentations, Speakers Bureau, manuscript writing, or educational events from Daiichi Sankyo Co, Ltd, Nippon Boehringer Ingelheim Co, Ltd, Bristol-Meyers Squibb, KK; Bayer Yakuhin, Ltd, Pfizer Japan, Inc, Ono Pharmaceutical Co Ltd, and Medtronic Japan Co, Ltd. Dr Boriani has received small speaker fees from Boston Scientific, Bayer, Boehringer, Bristol Myers Squibb, Janssen, and Sanofi, outside the submitted work. Dr Lip is a consultant and speaker for BMS/Pfizer, Boehringer Ingelheim, Anthos, and Daiichi Sankyo Co, Ltd; no fees were received personally. All other authors have reported that they have no relationships relevant to the contents of this paper to disclose.
