# Supplementary material for: External Validation of COOL-AF Scores in the Asian Pacific Heart Rhythm Society Atrial Fibrillation Registry
Source: JACC Asia. 2023 Nov 14;4(1):59–69. doi: 10.1016/j.jacasi.2023.09.011 (PMC10782403; doi:10.1016/j.jacasi.2023.09.011)

**Supplemental Figure 1.** Decision curve analysis according to COOL-AF scores and CHA_2_DS_2_-VASc or HAS-BLED in APHRS


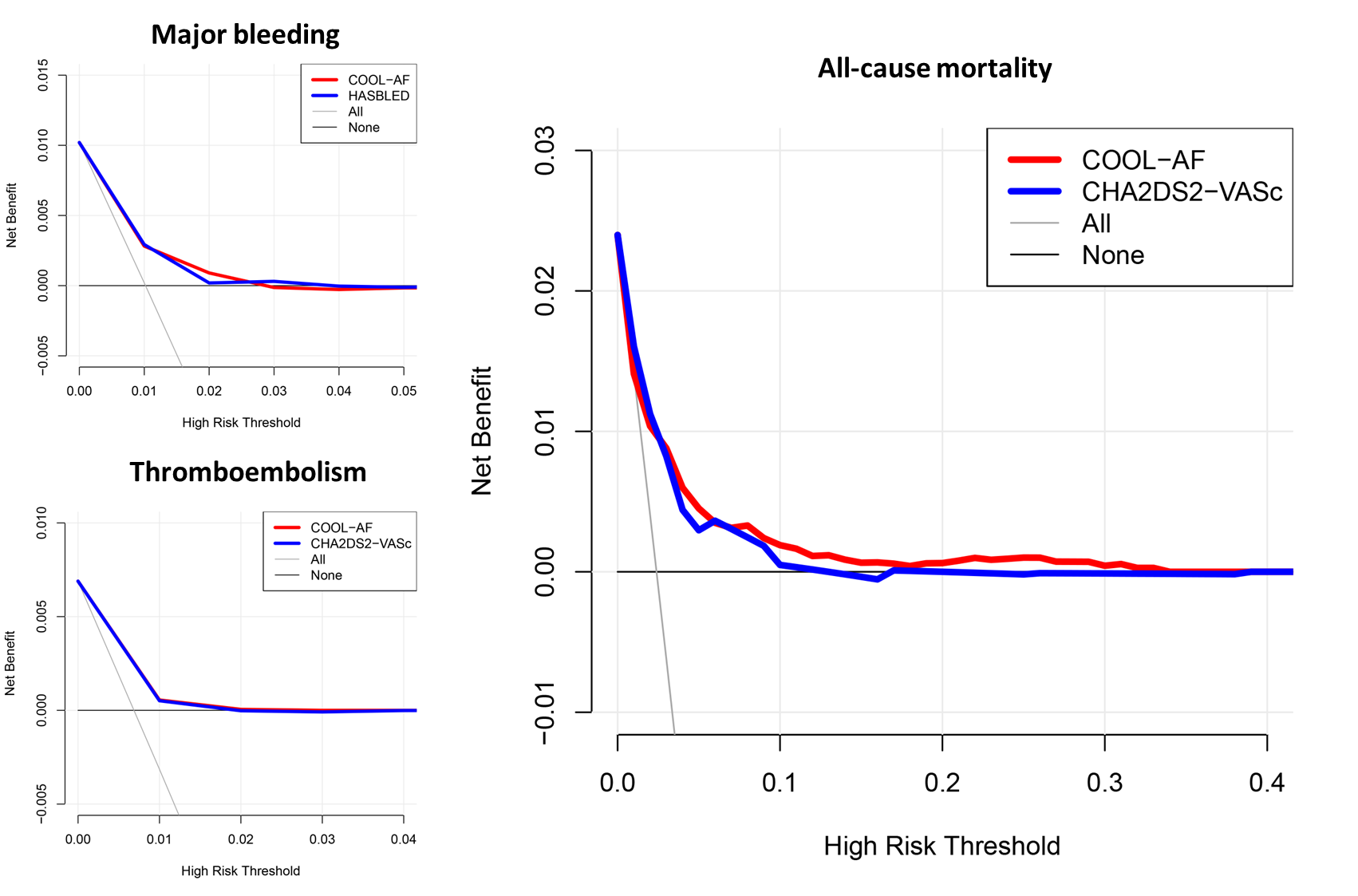


**Supplemental Figure 2.** Decision curve analysis according to COOL-AF scores and CHA_2_DS_2_-VASc or HAS-BLED in EORP-AF


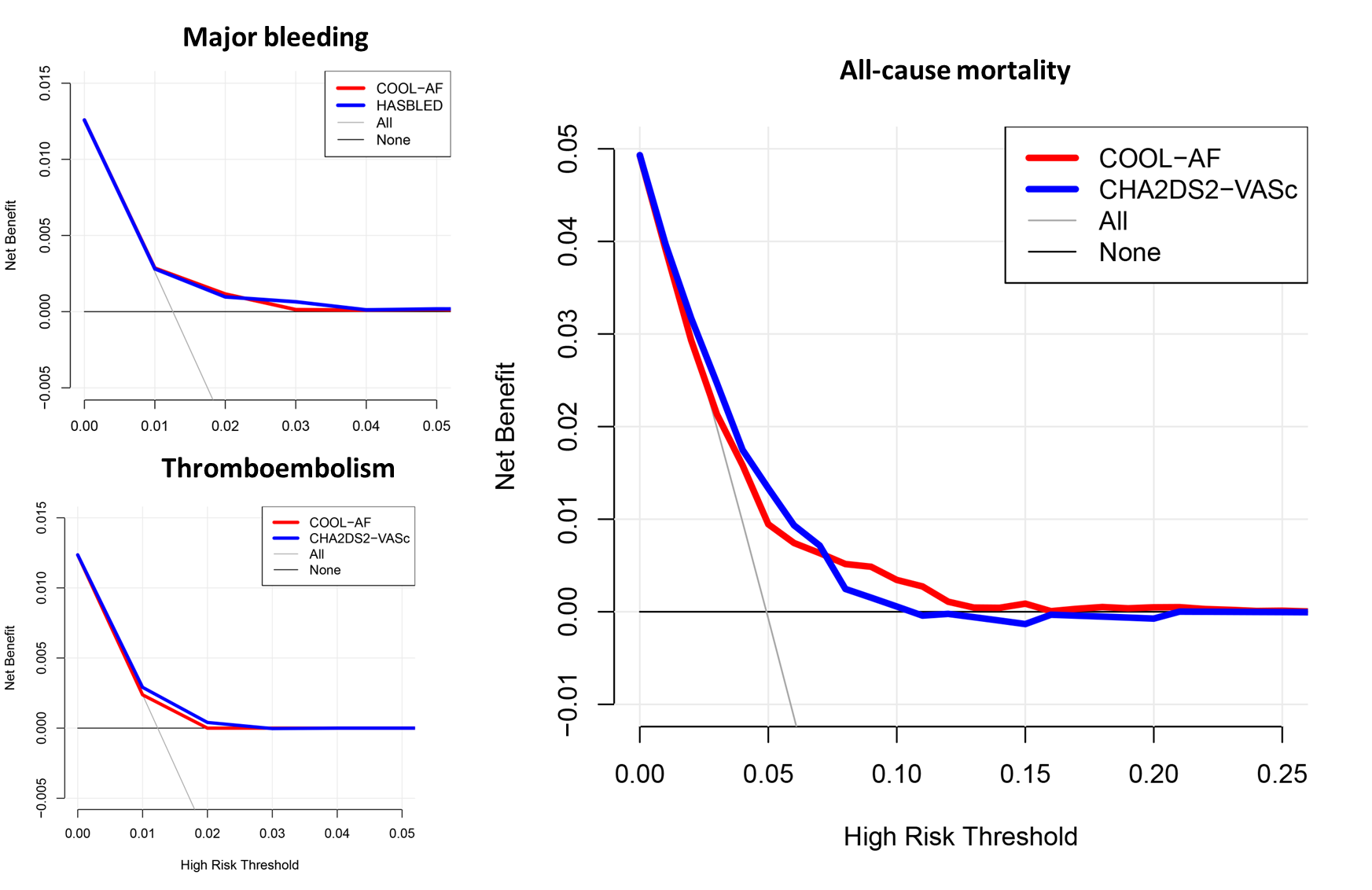

Supplement: Supplemental Figures 1 and 2 [file mmc1.docx]
